# Estimating Potential Benefits to Neurocognition with Proton Therapy in Adults with Brain Tumors

**DOI:** 10.14338/IJPT-22-00024.1

**Published:** 2023-03-16

**Authors:** Mariana Petruccelli, Amy Parent, Michael Holwell, Hitesh Dama, Grace Tsui, Zhihui Amy Liu, Derek S. Tsang

**Affiliations:** 1 Radiation Medicine Program, Princess Margaret Cancer Centre, University Health Network, Toronto, ON, Canada; 2 Department of Biostatistics, Princess Margaret Cancer Centre, University Health Network, Toronto, ON, Canada

**Keywords:** brain neoplasms, cognition, proton therapy, radiation therapy planning, computer-assisted, survivorship

## Abstract

**Purpose:**

Photon radiation therapy (RT) is important in the treatment of many brain tumors but can negatively affect neurocognition. Proton therapy (PT) can reduce doses to normal brain structures. We compared photon and proton plans to estimate the potential benefit in cognition if the patient were treated with PT.

**Materials and Methods:**

We analyzed 23 adult patients with proton and photon plans for the treatment of a primary brain tumor. Cognitive outcomes were predicted using converted equivalent dose (EQD2) with an α/β ratio of 3 to left temporal lobe and normal brain tissue. Risks of cognitive decline on 2 specific tests, the Controlled Oral Word Association Test (COWAT [letter S], a test of verbal fluency) and the Wechler Adult Intelligence Scale (WAIS-IV Coding Test, a test of processing speed) were derived from a previously published model.

**Results:**

Dose reductions to left temporal lobe and normal brain tissue translated into lower estimated probabilities of impairment in specific neurocognitive test scores after PT. With a mean dose reduction from 1490 to 1092 cGy in EQD2 to the left temporal lobe (*P* < .001), there was reduction in probability of impairment in the COWAT (Letter S) test from 6.8% to 5.4%. Similar results were seen with the normal brain (750 to 451 cGy in EQD2, *P* < .001), with reduction in probability of impairment in the WAIS-IV Coding test from 5% to 4.1%. Other structures experiencing dose reduction with PT included each cochlea, posterior fossa, each temporal lobe, and each hippocampus.

**Conclusion:**

We confirmed an association between PT and lower doses to brain substructures, which is expected to result in a modest decrease in probability of impairment in neurocognitive test scoring. These findings should be confirmed in prospective cohorts of patients treated with PT.

## Introduction

Patients with lower-grade primary brain tumors often have good outcomes and survival, including the possibility of long-term (>10 year) survival. Among these tumors are astrocytomas and oligodendroglioma. Combined, these tumors make up an estimated 20% to 25% of all primary brain tumors [1]. Other tumors with excellent prognosis include meningiomas, craniopharyngiomas, ependymomas, and pilocytic astrocytomas.

Radiation therapy (RT) is known to negatively affect neurocognition in patients with brain tumors [2, 3]. In a study by Klein et al, low-grade glioma (LGG) patients who received RT did not do as well in neurocognitive testing as those who did not receive RT, with patients who received large doses per fraction at greatest risk [4]. In an analysis of 20 patients treated at the Mayo Clinic with 50.4 Gy or 64.8 Gy RT for LGG, the authors found that while most patients did not experience neurocognitive decline, individuals that did lose cognitive function tended to be in the high-dose group, suggesting a dose-response relationship (higher doses cause more damage) [5].

Currently, patients in Canada are being treated with photon RT as the standard-of-care. Recently, proton RT has become available outside the country [6, 7]. This treatment allows radiation to be precisely delivered at the desired depth of tissue penetration while minimizing the dose of radiation to healthy surrounding tissue [8, 9]. Proton therapy (PT) is able to reduce the dose to normal brain structures as compared with photon RT [9, 10]; however, the actual clinical and neurocognitive benefit is not well known, given the paucity of long-term follow-up data in adult patients treated with PT. Doctors, patients, and families will desire the ability to critically evaluate and quantitatively estimate the benefits of proton over photon RT prior to embarking on travel to a distant proton center.

The goal of this study was to compare dosimetry between photon and proton RT plans to the brain, and to estimate the magnitude of benefit in dosimetry and cognition if the patient were treated with proton beam therapy using published models from the radiation oncology literature.

## Materials and Methods

Patients treated using fractionated photon RT for primary brain tumors between 2008 and 2018 at the Princess Margaret Cancer Centre and whose radiation dosimetry information was accessible, retrievable, and exportable from the treatment planning system (Pinnacle; Philips, Amsterdam, Netherlands) were originally eligible for our study. Eligible patients in this original cohort must also have had at least 2 neuropsychological evaluations in our supportive care department, results of which were reported as part of a separate study [11]. We identified 11 patients meeting these original criteria. As part of an expanded cohort of 12 additional patients, individuals treated up until 2022 with available proton and photon plans were included to increase the study sample size (see Supplementary Information). Patients were treated with intensity-modulated radiation therapy (original cohort, n = 11), treated/planned with volumetric arc therapy (expanded cohort, n = 8), or treated with proton therapy (expanded cohort, n = 4). This study was approved by the University Health Network research ethics board (18-5563).

Proton plans were generated to be compared dosimetrically with these patients' clinical photon plans. Both clinical photon plans and *in silico* proton plans were created by a dosimetrist with proton planning expertise. This planning was done using RayStation (RaySearch Laboratories, Stockholm, Sweden), which is able to simulate a pencil beam scanning system capable of energies up to 250 MeV with a 3-mm spot size. The proton plans were planned with clinical target volume (CTV) robustness to 3 mm of movement and 3% change in the computed tomography calibration curve. Photon plans were planned to cover the planning target volume (PTV) using volumetric arc therapy. The homogeneity goal for target coverage was 95% to 105%. All plans were evaluated by a specialist radiation oncologist.

Dosimetric data (mean and D50) to prespecified targets and organs-at-risk were collected. Paired *t* tests were used to determine statistical significance of the dose difference to each individual brain substructure between the proton and photon dose distributions; *P* < .05 was defined as statistically significant.

Cognitive outcomes were predicted using converted equivalent dose (EQD2) with an α/β ratio of 3 to left temporal lobe, and normal brain (brain not including the CTV and the brainstem). These structures were chosen based on structures with a statistically significant decrease in dose exposure in the original cohort and were found to be clinically relevant for cognition based on a model for risk of neurocognitive decline reported previously by Haldbo-Classen et al [12]. In that study, models for cognitive function were created using a dataset of 78 primary brain tumor patients treated with conventionally fractionated RT across different histologies. Risks of cognitive decline were derived from data, figures, and model coefficients available from the the study by Haldbo-Classen et al [12]. Dose data from the present study were used to compare the photon and proton plans and to estimate probability of decline in test scores after treatment.

## Results

**Figure 1.**
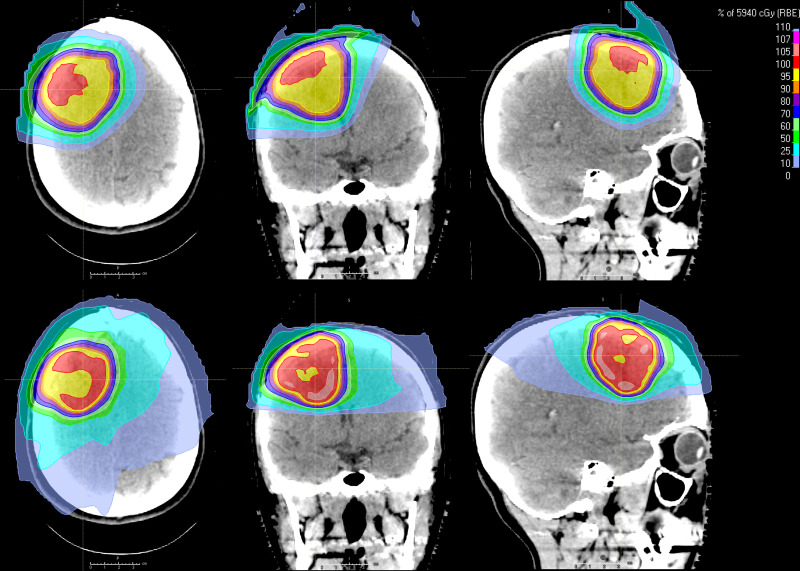
Comparison of proton (upper panels) and photon (lower panels) plans and isodose line distributions for a representative patient. Axial, coronal, and sagittal views are displayed from left to right.

We analyzed clinical photon plans and research proton plans of 23 patients with primary brain tumors; an example is shown in **Figure 1**. All patients were older than 18 years at the time of treatment, and most of them (21 patients, 91% of our cohort) received focal treatment with radiation only. The 2 patients (9%) who received treatment to the whole brain followed by a boost had only the boost plan analyzed. Patient, tumor, and treatment characteristics are shown in **Table 1**.

**Table 1.** Patient, tumor, and treatment characteristics.

**Patient characteristics**	**Value, N = 23**
Age at time of photon RT, median (range), y	42 (19-61)
Sex, n (%)	
Male	7 (30)
Female	16 (70)
Tumor type, n (%)	
Meningioma	9 (39)
Ependymoma	3 (13)
Craniopharyngioma	3 (13)
Solitary fibrous tumor	3 (13)
Medulloblastoma	1 (4)
Astrocytoma	1 (4)
Germinoma	1 (4)
Chondrosarcoma	1 (4)
Sinonasal carcinoma	1 (4)
Extent of surgery, n (%)	
None	6 (26)
Biopsy	2 (9)
Subtotal resection	10 (43)
Gross total resection	5 (22)
Location, n (%)	
Supratentorial	9 (39)
Skull base	11 (48)
Posterior fossa	3 (13)
Laterality, n (%)	
Left	10 (43)
Right	6 (26)
Midline	7 (30)
Chemotherapy, n (%)	
None	21 (91)
Temozolomide	1 (4)
Immunotherapy	1 (4)
RT dose, median (range), Gy	54 (15-70)
GTV, median (range), cm^3^	14.1 (0.85-90.0)
CTV, median (range), cm^3^	44.2 (2.1-296.2)

**Abbreviations:** RT, radiation therapy; GTV, gross tumor volume; CTV, clinical target volume.

**Figure 2.**
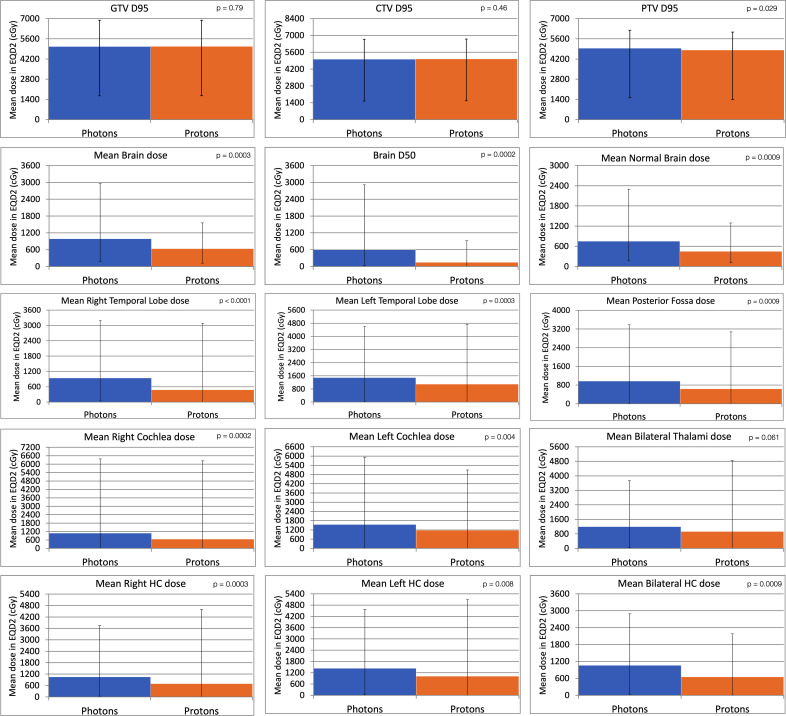
Comparison of the mean dose or D50 to tumor targets and normal brain structures. Whiskers represent maximum and minimum values. P values were calculated using paired t tests. Abbreviations: CTV, clinical target volume; Dxx, dose to xx% of the structure; GTV, gross tumor volume; HC, hippocampus; PTV, planning target volume.

When we compared photon and proton plans, there was reduction of the average mean dose to most of the analyzed structures with proton therapy as compared with the photon plans (**Figure 2**). Statistically significant reductions were found when comparing doses to the brain, posterior fossa, each cochlea, each temporal lobe, each hippocampus, and normal brain (**Figure 2**). Gross tumor volume (GTV) and CTV coverages were unchanged between proton and photon plans. Because proton plans were designed with robust planning to cover CTVs, the PTV coverage was slightly lower with proton therapy (*P* = .029).

When applied to the cognitive model, the dose reduction to left temporal lobe and normal brain translated to modestly reduced estimated probabilities of decline in specific neurocognitive test scores after treatment. Lower doses to left temporal lobe were associated with better scores in the Controlled Oral Word Association Test (COWAT) (letter S) test, which is a test of verbal fluency. Lower doses to normal brain were associated with better scores in the Wechsler Adult Intelligence Scale (WAIS)-IV Coding test, which is a test of processing speed. In our cohort, we saw a mean dose reduction from 1490 to 1092 cGy in EQD2 to the left temporal lobe (*P* = .0003), which translated to a reduction in the probability of those patients having lower test scores (impairment) in the COWAT (Letter S) test of 6.8% to 5.4% (**Table 2**). A similar result was seen with normal brain (750 to 451 cGy in EQD2, *P* = .0009), with the resulting reduction in probability of impairment in the WAIS-IV Coding test from 5% to 4.1% (**Table 2**). Whether these estimates would lead to detectable clinical improvement if the patients were actually treated with proton therapy needs to be investigated further with prospective studies.

**Table 2.** Reduction in mean dose and estimated probability of decline in cognitive test scores between photon and proton treatment.

**Structure**	**Average mean dose in EQD2 (cGy)**			**Mean probability of decline in test scores (%)**	
	**Photon**	**Proton**	***P*** **value**	**Photon**	**Proton**
Normal brain^a^	750	451	.0009	5.0	4.1
Left temporal lobe	1490	1092	.0003	6.8	5.4

a Defined as brain minus (clinical target volume and brainstem).

There is no proven correlation between lower doses to cochlea and improved test scores in adults, but it is known that hearing deficits in children can lead to difficulties in learning [13] and can affect their academic achievements and social life [14]. The current study shows that there was a statistically significant reduction in the dose to the right and left cochleae.

## Discussion

Patients with primary brain tumors often need treatment that includes RT [15]. Radiation can be very effective in treating these tumors but is often associated with the possibility of neurocognitive decline as a long-term toxicity [2–5], especially in patients diagnosed with tumors associated with good prognoses. Recent advances in imaging, RT techniques, and development of new treatment technologies have reduced the volume of normal tissue being irradiated, which could potentially reduce these late side effects [16]. In this study, we compared photon and proton plans and found that most brain substructures received less radiation if the treatment was delivered with protons, which would be expected to result in a modest reduction in the probability of neurocognitive decline associated with RT.

Different areas of the brain are responsible for different neurologic and cognitive functions. Neurocognitive toxicity represents a spectrum of different toxicities, and the time course of developing cognitive change can vary significantly [12, 17]. There are few studies that evaluate whether specific brain substructures were associated with neurocognitive decline after RT. Gondi et al [18] evaluated 18 patients who had had baseline and follow-up testing and found a dose-response relationship between radiation dose to the hippocampus and memory (ability to recall a list of words learned previously, also known as delayed recall). Ma et al also found a correlation between radiation dose to hippocampi and memory decline in patients treated with cranial RT and associated a D50% of the bilateral hippocampi receiving 22.1 Gy with a 20% chance of decline [19]. Looking to find similar connections between RT dose to specific left-sided brain substructures and neurocognitive decline, Haldbo-Classen et al showed that high RT doses to the left hippocampus and other left-side structures could result in impairments in verbal fluency, executive function, and processing speed [12]. Sekely et al specifically looked at patients with meningioma in a cross-sectional study and found associations with higher dose to the parieto-occipital region and slower visuomotor processing speed [20].

With the advent of PT and its possible benefit in reducing the incidence of long-term toxicities after RT, radiation oncologists and neuro-oncologists are increasingly interested in offering treatment with protons to patients who would benefit from this. However, because of the paucity of data and the high cost involved in the acquisition of a PT treatment unit [21], many countries, including Canada, continue to use photon RT only. Individual provinces and their respective ministries of health currently review requests for out-of-country proton therapy on a case-by-case basis to evaluate clinical indications and potential benefits of PT, based on available guidelines [22, 23]. In the evaluations, where clinical indications for PT are uncertain, comparative dosimetry has value to quantify the dosimetric benefits of proton therapy as compared with photon therapy [24, 25]. A proton versus photon comparative planning program from Australia has significantly increased the number of referrals for PT overseas since its beginning in 2016 [26]. A similar Proton Therapy Consultation Service exists for Canadian patients (http://protonsatuhn.ca/).

Our dosimetric study is limited by use of *in silico* comparative data only. In addition, the sample size was small; we tried to mitigate this by combining the original cohort population with an expanded cohort, for a total of 23 patients. Finally, although there were statistically significant decreases to dose to most of the evaluated brain structures, the estimated absolute probability cognitive decline was low, which meant that the absolute magnitude of cognitive benefit from treating a patient with PT was also low (but statistically significant).

In conclusion, we were able to confirm an association between proton treatment and lower doses to specific brain substructures, including the left temporal lobe and normal brain. Lower doses to these structures have been shown to result in reduced chances of decline and impairment in neurocognitive test scoring [12]. Although our findings are limited by the small number of patients analyzed in this cohort and its retrospective nature, this study should encourage the continued development of prospective proton therapy research in adult neuro-oncology and survivorship research, with a larger number of patients to confirm the expected benefits of proton therapy.
