# Supplementary material for: Estimating Potential Benefits to Neurocognition with Proton Therapy in Adults with Brain Tumors
Source: Int J Part Ther. 2023 Mar 16;9(4):261–8. doi: 10.14338/IJPT-22-00024.1 (PMC10166017; doi:10.14338/IJPT-22-00024.1)
Supplement: Supplementary file 1 [file ijpt-09-04-04_s01.docx]

**Supplementary Information**

There were 11 patients as part of the **original cohort**.

Photon plans

- 11 photon plans in this cohort were clinically treated and delivered to the patient.

Proton plans

- 11 proton plans were created specifically for this study, using methods described in the manuscript.

We identified 12 patients as part of an **expanded cohort**.

Photon plans

- 7 photon plans were clinically treated and delivered to the patient
- 4 photon plans were created specifically for this study, using institutional protocols for focal VMAT brain radiotherapy; these patients received proton therapy out-of-country
- 1 photon plan was created as part of a local comparative planning service ([www.protonsatuhn.ca](http://www.protonsatuhn.ca)).

Proton plans

- 5 proton plans were created specifically for this study, using methods described in this manuscript
- 4 proton plans were clinically treated and delivered to the patient, as part of our province’s out-of-country treatment program (<https://www.health.gov.on.ca/en/public/programs/ohip/outofcountry/proton.aspx>).
- 3 proton plans created as part of a local comparative planning service ([www.protonsatuhn.ca](http://www.protonsatuhn.ca))
